# Quantifying Regional Lung Deformation Using Four-Dimensional Computed Tomography: A Comparison of Conventional and Oscillatory Ventilation

**DOI:** 10.3389/fphys.2020.00014

**Published:** 2020-02-20

**Authors:** Jacob Herrmann, Sarah E. Gerard, Wei Shao, Monica L. Hawley, Joseph M. Reinhardt, Gary E. Christensen, Eric A. Hoffman, David W. Kaczka

**Affiliations:** ^1^Roy J. Carver Department of Biomedical Engineering, University of Iowa, Iowa City, IA, United States; ^2^Department of Anesthesia, University of Iowa, Iowa City, IA, United States; ^3^OscillaVent, Inc., Iowa City, IA, United States; ^4^Department of Electrical and Computer Engineering, University of Iowa, Iowa City, IA, United States; ^5^Department of Radiology, University of Iowa, Iowa City, IA, United States; ^6^Department of Radiation Oncology, University of Iowa, Iowa City, IA, United States; ^7^Department of Internal Medicine, University of Iowa, Iowa City, IA, United States

**Keywords:** mechanical ventilation, ventilator-induced lung injury, respiratory mechanics, high-frequency oscillatory ventilation, multi-frequency oscillatory ventilation, computed tomography, image registration

## Abstract

Mechanical ventilation strategies that reduce the heterogeneity of regional lung stress and strain may reduce the risk of ventilator-induced lung injury (VILI). In this study, we used registration of four-dimensional computed tomographic (4DCT) images to assess regional lung aeration and deformation in 10 pigs under baseline conditions and following acute lung injury induced with oleic acid. CT images were obtained via dynamic axial imaging (Siemens SOMATOM Force) during conventional pressure-controlled mechanical ventilation (CMV), as well as high-frequency and multi-frequency oscillatory ventilation modalities (HFOV and MFOV, respectively). Our results demonstrate that oscillatory modalities reduce intratidal strain throughout the lung in comparison to conventional ventilation, as well as the spatial gradients of dynamic strain along the dorsal-ventral axis. Harmonic distortion of parenchymal deformation was observed during HFOV with a single discrete sinusoid delivered at the airway opening, suggesting inherent mechanical nonlinearity of the lung tissues. MFOV may therefore provide improved lung-protective ventilation by reducing strain magnitudes and spatial gradients of strain compared to either CMV or HFOV.

## Introduction

Ventilator-induced lung injury (VILI) may inadvertently occur in critically ill patients receiving mechanical ventilation, due to the harmful stresses and strains associated with gas flows driven under positive pressure (Slutsky and Ranieri, 2013). Patients with injured, inflamed, and/or edematous lungs, such as those with the acute respiratory distress syndrome (ARDS), are especially at risk for VILI due to increased ventilation heterogeneity (Carrasco Loza et al., 2015). Maintaining normal levels of arterial oxygen and carbon dioxide tensions in patients with ARDS may be extremely difficult using conventional mechanical ventilation (CMV), since increasing the tidal volume or driving pressure to compensate for gas exchange deficiencies may be detrimental to the mechanically over-burdened lung (Acute Respiratory Distress Syndrome Network, 2000; Amato et al., 2015; Gattinoni et al., 2016a). The goal of lung-protective ventilation is to provide life-sustaining gas exchange without exacerbating existing injury within vulnerable parenchymal tissues.

In some instances, high-frequency oscillatory ventilation (HFOV) has been proposed as a rescue treatment for refractory hypoxemia in ARDS, given its theoretically ideal qualities for lung-protection: small tidal volumes that mitigate the risk of dynamic strain injury (i.e., volutrauma), and high mean airway pressures that prevent cyclic recruitment/derecruitment (i.e., atelectrauma) (Sklar et al., 2017). Despite an extensive history of scientific and clinical research over several decades, optimal strategies for HFOV initiation and management remain a subject of controversy (Malhotra and Drazen, 2013; Kneyber and Markhorst, 2016; Nguyen et al., 2016). As it is currently delivered, HFOV may not be an appropriate ventilatory modality in many patients with ARDS for several reasons. First, the use of high mean airway pressures may result in hemodynamic compromise due to impaired venous return and low cardiac output (Meade et al., 2017). In addition, high strain rates within the lung tissues during HFOV may also contribute to VILI, by increasing the mechanical power dissipated across the parenchyma (Gattinoni et al., 2016b; Protti et al., 2016; Herrmann et al., 2019b). Finally, high frequency oscillatory flows are distributed in a heterogeneous and frequency-dependent manner, predisposing overventilated regions to excess mechanical strain, and underventilated regions to derecruitment and atelectasis (Amini and Kaczka, 2013; Herrmann et al., 2016).

Multi-frequency oscillatory ventilation (MFOV) has thus also been proposed as an alternative modality for lung-protective ventilation, by delivering multiple frequencies of oscillatory flow and pressure simultaneously (Kaczka et al., 2015; Herrmann et al., 2019b). MFOV has been shown to improve gas exchange and mechanical function compared to traditional “single-frequency” HFOV (Kaczka et al., 2015; Herrmann et al., 2018). Its postulated mechanism of benefit relies on the frequency-dependence of ventilation distribution and gas transport during conventional and oscillatory modes of ventilation (Herrmann et al., 2018), which can be exploited by using flow waveforms with enhanced harmonic content. Given such frequency-dependence of gas flow throughout the airway tree, mechanically disparate regions of an injured lung may thus selectively filter out “less desirable” frequency components of a broadband oscillatory waveform delivered at the airway opening. The corresponding homogenization of intrapulmonary gas transport with MFOV may then contribute to enhanced gas exchange, and simultaneously distribute the mechanical burden of ventilation more uniformly throughout the lung. Nonetheless, specific experimental evidence to support such a mechanism is lacking, and it remains to be seen whether MFOV improves the regional distribution of parenchymal strain throughout the lung compared to CMV or traditional HFOV.

The purpose of this study was to characterize the distribution of regional intratidal lung deformation during conventional and oscillatory modes of ventilation in pigs under baseline conditions and following acute lung injury with oleic acid. We hypothesized that such variations would be less heterogeneous during MFOV, compared to CMV or traditional single-frequency HFOV. Specifically, we quantified intratidal variations in dynamic regional lung aeration, volumetric strain, and volumetric strain rate during CMV, HFOV, and MFOV using frequency-selective four-dimensional computed tomographic (4DCT) image reconstruction (Herrmann et al., 2017) and registration (Zhao et al., 2016).

## Materials and Methods

### Animal Preparation

All experimental procedures were approved by the University of Iowa Institute for Animal Care and Use Committee (Protocol Number 5061428). Ten healthy pigs were used in this study, weighing between 9 and 13 kg. Following intramuscular injection of 2.2 mg kg^−1^ telazol, 1.1 mg kg^−1^ ketamine, and 1.1 mg kg^−1^ xylazine, general anesthesia was induced during spontaneous breathing with inhaled isoflurane delivered via nosecone. Each pig was then intubated with a cuffed endotracheal tube (4.5–5.5 mm inner diameter), and mechanically ventilated (Uni-Vent Eagle Model 754, ZOLL Medical Corporation, Chelmsford, MA). Capnography, peripheral oxygen saturation (*S*_p_O_2_), and electrocardiogram waveforms were obtained using a Philips patient monitor equipped with the M3001A measurement module (Philips Healthcare, Andover, MA). Surgical incision into the neck was performed to allow cannulation of the internal jugular vein and carotid artery, as well as relocation of the endotracheal tube into an incision in the trachea just below the larynx. Endotracheal tubes were manually shortened (to final length 12–15 cm depending on tube diameter) prior to tracheostomy, to reduce apparatus deadspace. Anesthesia was maintained using an intravenous infusion of propofol (7–9 mg kg^−1^ hr^−1^), and muscular relaxation was achieved using intermittent intravenous boluses of either rocuronium (1–2 mg kg^−1^) or pancuronium (0.01–0.15 mg kg^−1^).

Lung injury was induced by slow infusion of 0.08 cm^3^ kg^−1^ oleic acid into the internal jugular vein over 15 min. Maturation of injury required ~90 min, and was confirmed by a ratio of arterial oxygen tension to inspired oxygen fraction (*P*_a_O_2_:*F*_i_O_2_) <300 mmHg during ventilation with 5 cmH_2_O of positive end-expiratory pressure (PEEP). Additional oleic acid (0.04 cm^3^ kg^−1^) was administered if *P*_a_O_2_:*F*_i_O_2_ remained >300 mmHg 90 min after the initial dose. If necessary, normal systemic arterial blood pressure (systolic/diastolic ≥ 90/60 mmHg) was maintained with intravenous crystalloid solutions and intermittent doses of phenylephrine (1–2 μg kg^−1^).

### Experimental Protocol

All measurements and ventilation modalities were performed under baseline conditions and following lung injury. Each subject was mechanically ventilated with CMV, HFOV, and MFOV in random order over 30-min intervals, using a FabianHFO hybrid oscillator/ventilator (Acutronic Medical Systems AG, Switzerland). Mean airway pressure (${\bar{P}}_{\text{aw}}$) was set to 12 cmH_2_O for all modalities. During baseline conditions, *F*_i_O_2_ was set to 40%, but was increased to maintain *S*_p_O_2_ ≥ 90% following lung injury. Pressure-controlled CMV was delivered at rates between 20 and 32 min^−1^ (0.33 to 0.53 Hz), with inspiratory:expiratory ratio of 1:2. Single-frequency HFOV was delivered at 5 Hz, while MFOV was delivered using a combination of 5, 10, 15, and 20 Hz superimposed sinusoids with uniform flow amplitudes (Kaczka et al., 2015). Example ventilator waveforms are provided in Supplementary Figures S-1, S-2, S-3. Sampling frequency for recorded ventilator waveforms was 200 Hz. Ventilator driving pressure or pressure amplitudes were adjusted to obtain arterial CO_2_ tension (*P*_a_CO_2_) in the target range of 30–60 mmHg. Each 30-min experimental interval was followed by an arterial blood gas analysis and 4DCT scan sequence, without interrupting mechanical ventilation (Herrmann et al., 2017). Between each experimental ventilation interval, a 15-min wash-out period of CMV and a 30-s recruitment maneuver to 35 cmH_2_O of airway pressure were used to restore a control mechanical and physiological state. After completion of the experimental protocol, subjects were euthanized with an intravenous solution of pentobarbital sodium and phenytoin sodium (1 mL + 0.2 mL kg^−1^).

### Assessment of Gas Exchange and Mechanics

Oxygenation was assessed using the oxygenation index (OI), defined as (Ortiz et al., 1987):

(1) $$\begin{array}{l} \text{OI}=\frac{{\bar{P}}_{\text{aw}}\cdot F_{\text{i}}{\text{O}}_{2}}{P_{\text{a}}{\text{O}}_{2}} \end{array}$$

The efficiency of CO_2_ elimination during each modality was assessed using a ventilatory cost function (*V*_C_), defined as (Kaczka et al., 2015):

(2) $$\begin{array}{l} V_{\text{C}}=\frac{V_{\text{rms}}^{2}\cdot P_{\text{a}}{\text{CO}}_{2}}{\text{Wt}} \end{array}$$

where *V*_rms_ is the root mean-square of the volume waveform *V*(*t*) delivered to the airway opening:

(3) $$\begin{array}{l} V_{\text{rms}}=\sqrt{\frac{1}{T}\int_{0}^{T}{\left[V\left(t\right)-\bar{V}\right]}^{2}\text{d}t}. \end{array}$$

*T* denotes the time duration over which the integration in Equation (3) is performed (i.e., one breath for CMV or one second during HFOV/MFOV), and $\bar{V}$ is the mean of the volume waveform over the interval 0 to *T*.^1^ Peak-to-peak range of volume (*V*_pp_) was computed as the difference between the largest and smallest values of time-varying volume:

(4) $$\begin{array}{l} V_{\text{pp}}=\underset{t}{\text{max }}V\left(t\right)-\underset{t}{\text{min }}V\left(t\right). \end{array}$$

Dynamic respiratory system elastance (*E*_rs_) during pressure-controlled CMV was computed as the quotient of driving pressure (Δ*P*_aw_) and tidal volume:

(5) $$\begin{array}{l} E_{\text{rs}}=\Delta P_{\text{aw}}\cdot V_{\text{pp}}^{-1} \end{array}$$

where:

(6) $$\begin{array}{l} \Delta P_{\text{aw}}=\underset{t}{\text{max }}P_{\text{aw}}\left(t\right)-\underset{t}{\text{min }}P_{\text{aw}}\left(t\right). \end{array}$$

Measurements of respiratory impedance (*Z*_rs_) were obtained under baseline conditions and after maturation of lung injury. A pseudorandom waveform consisting of nine sinusoids ranging in frequency from 0.078 to 8.9 Hz was generated by a digital-to-analog converter (NI USB-6356, National Instruments, Austin, TX), low-pass filtered at 12 Hz (858L8B-2, Frequency Devices, Haverhill, MA), and used as the input driving signal to a custom servo-controlled pneumatic pressure oscillator (Kaczka and Lutchen, 2004). Airway pressure was measured with a transducer placed at the proximal end of the endotracheal tube (Celesco LCVR-0050, Canoga Park, CA), while flow was measured using a screen pneumotach (4700A, Hans Rudolph, Shawnee, KS) coupled to a differential pressure transducer (Celesco LCVR-0002, Canoga Park, CA). The airway pressure and flow signals were sampled at 40 Hz over ~90 s during the application of forced oscillations, allowing for three repetitions of the 25.6-s periodic control signal. Each *Z*_rs_ spectrum and its corresponding coherence function (γ^2^) was computed using the Welch periodogram technique (Welch, 1967), with rectangular windowing and 80% overlap. The complex values of *Z*_rs_ were evaluated only at the nine frequencies used in the control signal (Suki and Lutchen, 1992). Corresponding resistance *R*_rs_ and reactance *X*_rs_ spectra were defined by the real and imaginary parts of *Z*_rs_, respectively. The resonant frequency *f*_res_ was estimated from the zero-crossing of *X*_rs_ using spline interpolation. Each *Z*_rs_ spectrum was then characterized by a 4-element constant-phase model consisting of parameters for Newtonian airway and chest wall resistance (*R*), airway inertance (*I*_aw_), tissue hysteresivity (η), and tissue elastance (*H*):

(7) $$\begin{array}{l} {\hat{Z}}_{\text{rs}}\left(f\right)=R+j2\pi fI_{\text{aw}}+\frac{\left(\eta -j\right)H}{{\left(2\pi f\right)}^{\alpha }} \end{array}$$

where Ẑ_rs_ denotes the model-predicted *Z*_rs_ and $\alpha =\left(\frac{2}{\pi }\right)\tan^{-1}\left(\frac{1}{\eta }\right)$. Model parameters were estimated by constrained nonlinear gradient descent technique (MATLAB, Natick, MA).

### Image Acquisition and Processing

CT scans were acquired using a Siemens SOMATOM Force (Siemens Healthineers, Germany) in an axial scanning mode, with 5.76 cm of axial coverage and 0.6 mm slice thickness. Subjects were continuously scanned at 80 kVp tube voltage and 150 mA tube current, with 250 ms scanner rotation period. Each scan lasted a total duration of 30 s, resulting in a radiation exposure of 345 mGy and generating a continuous set of projection images. The 4DCT image sequences were reconstructed by retrospective binning of x-ray projection data according to mechanical ventilation phase using a frequency-selective reconstruction algorithm (Herrmann et al., 2017), yielding between 13 and 21 volumetric images per 4DCT sequence with isotropic 0.6 mm spatial resolution. The corresponding temporal sampling frequencies for 21-phase image sequences were 7 Hz during CMV with fundamental 0.33 Hz, and 105 Hz during HFOV or MFOV with fundamental 5 Hz. Each sequence was periodic in the temporal (i.e., ventilation phase) dimension, such that the choice of “initial” image in the sequence was arbitrary. Each volumetric image in the temporal sequence was labeled *I*_*n*_(**x**_*n*_), where *n* indexes the number of images in the sequence 0 through *N* − 1, and **x**_*n*_ is a vector representing 3-dimensional spatial position. Voxels corresponding to spatial positions within the lungs were identified by a fully automated segmentation algorithm using a deep convolutional neural network (Gerard et al., 2018, 2020), generating a distinct lung mask *M*_*n*_(**x**_*n*_) for each image phase. The neural network was trained using manually segmented lungs in CT images obtained from multiple datasets of experimental lung injury models, including a subset of images from the current study.

The periodic motion of respiratory structures was estimated using a deformable 4D image registration technique. This procedure produced *N* 3-dimensional transformation functions denoted as ϕ_*n*_(**X**) = **x**_*n*_, where **X** is a vector representing 3-dimensional spatial position within the reference frame of a phase-averaged image *I*_avg_(**X**), and **x**_*n*_ represents spatial position in image *I*_*n*_(**x**_*n*_). Image *I*_avg_(**X**) was used as the target for groupwise image registration of the images *I*_*n*_(**x**_*n*_), *n* = 0…*N* − 1. Thus, ϕ_*n*_(**X**) = **x**_*n*_ is a spatial mapping from the coordinate system of *I*_avg_(**X**) to that of *I*_*n*_(**x**_*n*_), such that a deformed image *I*_*n* → avg_(**X**) was generated with respiratory structures aligned to the target image as:

(8) $$\begin{array}{l} I_{n\to \text{avg}}\left(\text{X}\right)=I_{n}\left(\phi_{n}\left(\text{X}\right)\right) \end{array}$$

**Figure 1:**
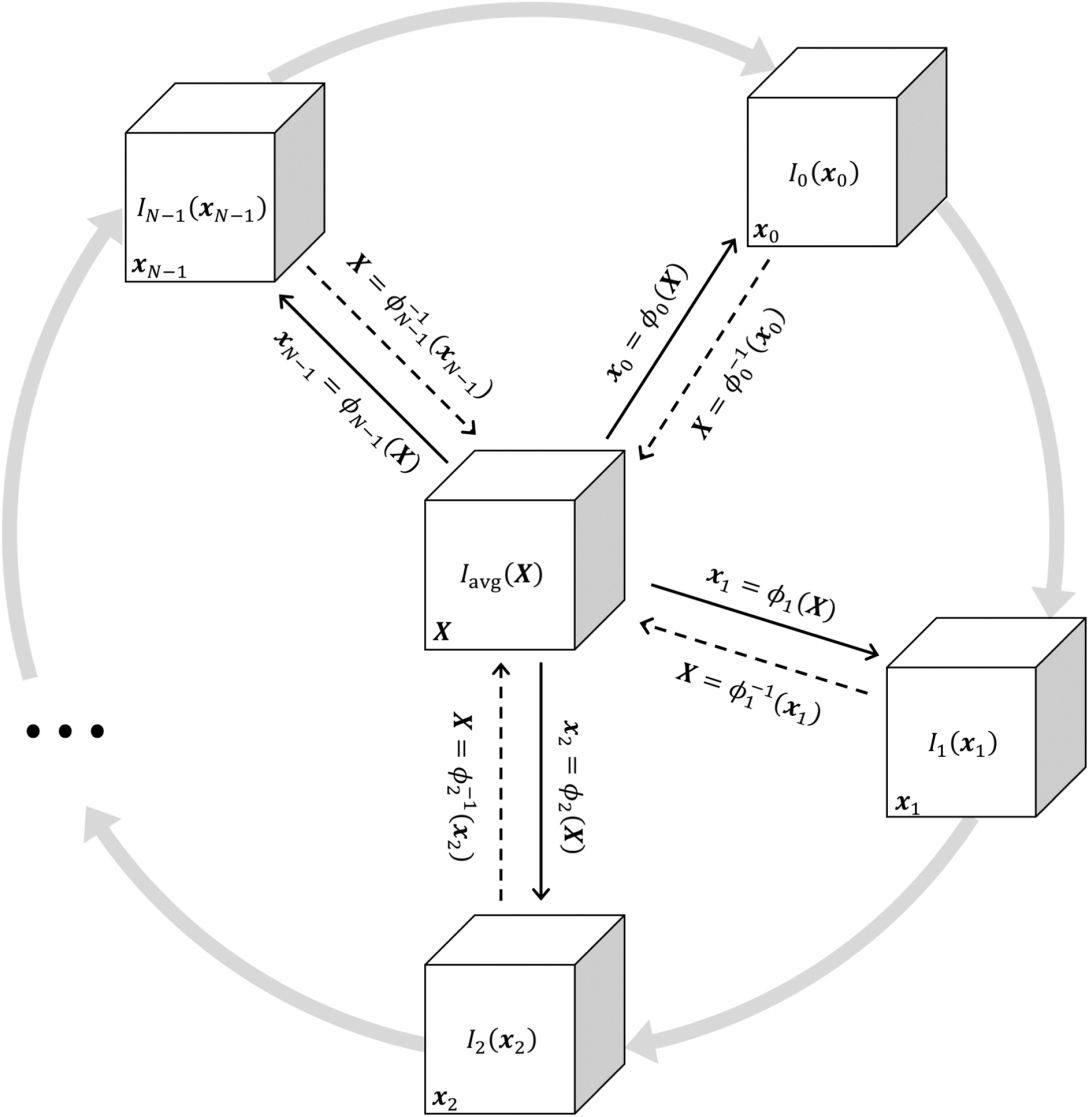
Schematic illustrating the jointly estimated 3D transformations ϕ_*n*_(**X**) = **x**_*n*_ mapping between all images in the periodic 4DCT image sequence *I*_*n*_(**x**_*n*_) and the target average image *I*_avg_(**X**). Solid black arrows indicate the transformation from the target reference frame **X** to the general reference frame **x**_*n*_. Dashed black arrows indicate the inverse transformation. Gray arrows represent the periodic sequence of *N* ventilatory phases indexed from 0 to *N* − 1. Any *I*_*n*_(**x**_*n*_) may be deformed to achieve structural alignment with any other *I*_*m*_(**x**_*m*_) by applying a sequence of two transformations ${\text{x}}_{n}=\phi_{n}\left(\phi_{m}^{-1}\left({\text{x}}_{m}\right)\right)$, warping **x**_*m*_ through **X**.

Figure 1 shows a schematic of the transformations mapping between all images in the periodic sequence and *I*_avg_(**X**). The transformations ϕ_*n*_(**X**) were coupled together using 4-dimensional cubic B-splines and jointly estimated to ensure smoothness across both spatial and temporal dimensions (Metz et al., 2011). The parameters of the transformation function were iteratively adjusted using the Elastix library (Klein et al., 2010) to minimize the sum of squared tissue volume differences (SSTVD) between each *I*_*n* → avg_(**X**) and *I*_avg_(**X**), such that each deformed image preserved the volume of tissue contained within each voxel (Gorbunova et al., 2008; Yin et al., 2009; Zhao et al., 2016). Thus, changes in CT voxel density due to variations in fractional gas content were adjusted using this similarity cost function. Following registration, ϕ_*n*_(**X**) and its inverse mapping $\phi_{n}^{-1}\left({\text{x}}_{n}\right)$ were used to align images between arbitrary phases. Deformed images were denoted as *I*_*n*→*m*_(**x**_*m*_), indicating the *n*th image deformed into the *m*th spatial reference frame:

(9) $$\begin{array}{l} I_{n\to m}\left({\text{x}}_{m}\right)=I_{n}\left(\phi_{n}\left(\phi_{m}^{-1}\left({\text{x}}_{m}\right)\right)\right) \end{array}$$

**Figure 2:**
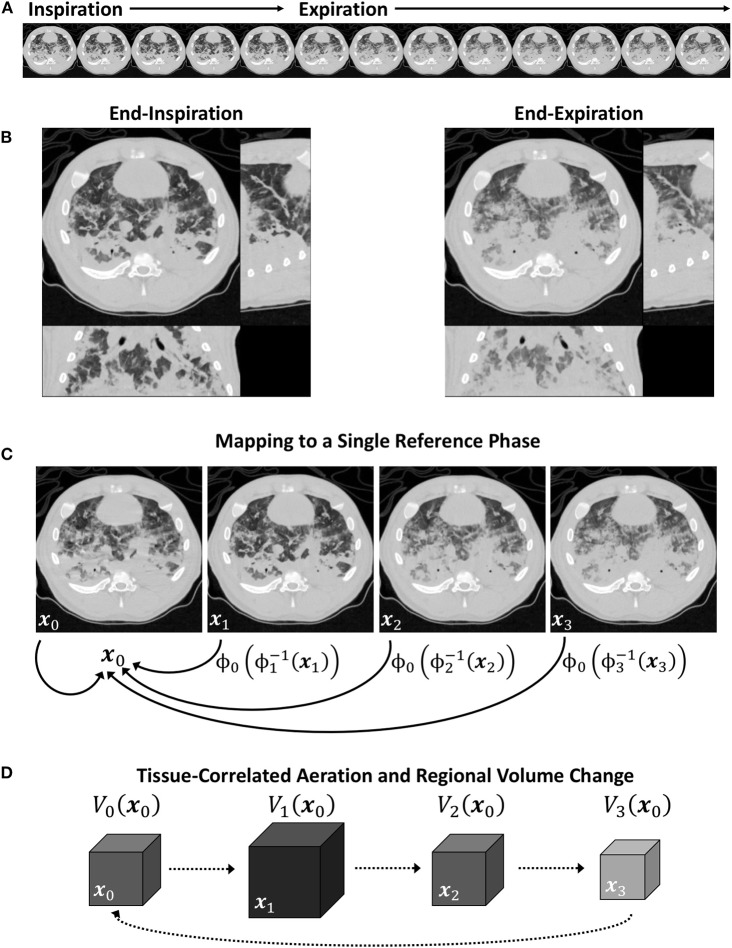
Schematic of a periodic 4DCT image sequence during conventional mechanical ventilation **(A)**, with inspiratory and expiratory phases **(B)**. Image registration is used to map each image in the sequence to a single reference phase **(C)**, after which intratidal variations in aeration and regional volume may be associated to specific regions of lung tissue **(D)**.

For the trivial case of *m* = *n*, Equation (9) simplifies to *I*_*n*→*n*_(**x**_*n*_) = *I*_*n*_(**x**_*n*_). Conceptually, aligning images from any two phases can be achieved by warping one spatial reference frame to another, passing through the target reference frame **X** (see Figure 1). Using this approach, all images were warped to align structures with a single, arbitrarily selected reference phase at *m* = 0. Phasic variations in regional aeration and strain could then be associated with the tissue contained within the region of interest at the reference phase. Figure 2 schematizes the use of a single reference phase to align respiratory structures across several ventilatory phases, thus tracking tissue-correlated changes in aeration and regional volume. Changes in regional aeration were then assessed by the range of voxel intensity values in the deformed image:

(10) $$\begin{array}{l} \Delta I\left({\text{x}}_{0}\right)=\underset{n}{\text{max }}I_{n\to 0}\left({\text{x}}_{0}\right)-\underset{n}{\text{min }}I_{n\to 0}\left({\text{x}}_{0}\right) \end{array}$$

while changes in regional volumetric strain were estimated using the Jacobian matrix ($J_{n\to m}$) of transformation spatial derivatives:

(11) $$\begin{array}{l} J_{n\to m}\left({\text{x}}_{m}\right)=\nabla_{{\text{x}}_{m}}\left(\phi_{n}\left(\phi_{m}^{-1}\left({\text{x}}_{m}\right)\right)\right) \end{array}$$

where ∇_**x**_*m*__ is the spatial gradient operator. Note that $J_{n\to m}$ is expressed in the *m*th spatial reference frame and describes the gradient of the “pullback” transformation $\phi_{n}\left(\phi_{m}^{-1}\left({\text{x}}_{m}\right)\right)$. This pullback transformation is interpreted as “pulling back” point **x**_*n*_ in ventilation phase *n* to the corresponding point **x**_*m*_ in phase *m*. Regional volume changes relative to the reference phase (i.e., *m* = 0) were computed by the determinant of $J_{n\to m}$:

(12) $$\begin{array}{l} \frac{V_{n}\left({\text{x}}_{0}\right)}{V_{0}\left({\text{x}}_{0}\right)}=|J_{n\to 0}\left({\text{x}}_{0}\right)| \end{array}$$

where *V*_*n*_(**x**_0_) corresponds to the phase-varying volume of a region which, in the reference phase 0, occupies a single voxel centered at position **x**_0_. Accordingly, *V*_0_(**x**_0_) is everywhere equal to the volume of a single voxel δ*V*, determined by the image spatial resolution. Thus, Equation (12) may be simplified to:

(13) $$\begin{array}{l} V_{n}\left({\text{x}}_{0}\right)=\delta V\cdot |J_{n\to 0}\left({\text{x}}_{0}\right)| \end{array}$$

**Figure 3:**
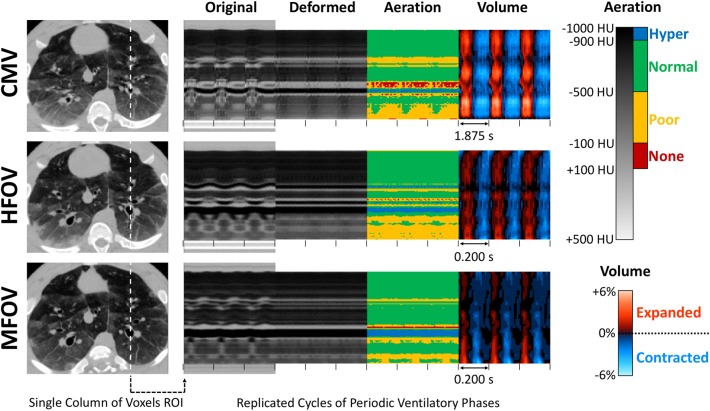
Four-dimensional image registration was used to deform images such that respiratory structures were aligned to a single reference phase. After registration, regional aeration and volume change throughout the lung were assessed by the variations in deformed image voxel value and determinant of the Jacobian matrix of the deformation. This technique was applied during conventional mechanical ventilation (CMV), high-frequency oscillatory ventilation (HFOV), and multi-frequency oscillatory ventilation (MFOV). Ventilatory cycle duration is indicated by spacing between tick marks. In this example, each ventilatory cycle corresponds to a sequence of images representing 21 distinct ventilatory phases. Note that multiple cycles of each ventilation waveform are shown for clarity, however all cycles are identical replicates. See also Supplementary Animations S-1, S-2.

Note that $|J_{n\to 0}|<1$ when the corresponding region deflates relative to the reference phase, $|J_{n\to 0}|>1$ when it expands, and $|J_{n\to 0}|=1$ when there is no volume change (i.e., isovolumetric deformation). Figure 3 shows an example image sequence for each ventilation modality in one injured subject, demonstrating phase-varying aeration *I*_*n* → 0_(**x**_0_) and volume change *V*_*n*_(**x**_0_) for each voxel in a sample region of interest in **x**_0_. Animated image sequences for one subject under baseline and injured conditions are also provided (Supplementary Animations S-1, S-2). Phase-varying strain (ε) for each lung region with respect to its respective minimum inflation state was defined as:

(14) $$\begin{array}{l} \varepsilon_{n}\left({\text{x}}_{0}\right)=\frac{V_{n}\left({\text{x}}_{0}\right)-\underset{n}{\text{min }}V_{n}\left({\text{x}}_{0}\right)}{\underset{n}{\text{min }}V_{n}\left({\text{x}}_{0}\right)} \end{array}$$

Changes in regional volumetric strain were assessed by the range of ε_*n*_(**x**_0_):

(15) $$\begin{array}{l} \Delta \varepsilon \left({\text{x}}_{0}\right)=\underset{n}{\text{max }}\varepsilon_{n}\left({\text{x}}_{0}\right)-\underset{n}{\text{min }}\varepsilon_{n}\left({\text{x}}_{0}\right) \end{array}$$

Since the minimum value of ε_*n*_(**x**_0_) is zero by definition, Equation (15) may be reduced to:

(16) $$\begin{array}{l} \Delta \varepsilon \left({\text{x}}_{0}\right)=\underset{n}{\text{max }}\varepsilon_{n}\left({\text{x}}_{0}\right) \end{array}$$

Similarly, regional volumetric strain rate ($\dot{\varepsilon }$) was computed by the temporal rate of change in ε_*n*_(**x**) using the forward difference scheme:

(17) $$\begin{array}{l} {\dot{\varepsilon }}_{n}\left({\text{x}}_{0}\right)=\frac{\varepsilon_{\left(n+1\right)\text{ mod }N}\left({\text{x}}_{0}\right)-\varepsilon_{n}\left({\text{x}}_{0}\right)}{\delta t} \end{array}$$

where δ*t* is the time difference between adjacent phases. The modulo operator is used to indicate the periodicity of ventilatory phase. The value of δ*t* is computed from the ventilatory fundamental frequency (*f*_0_) and the number of images in the 4DCT sequence (*N*):

(18) $$\begin{array}{l} \delta t=\frac{1}{f_{0}N} \end{array}$$

Finally, changes in regional volumetric strain rate were assessed by the range of ${\dot{\varepsilon }}_{n}\left({\text{x}}_{0}\right)$:

(19) $$\begin{array}{l} \Delta \dot{\varepsilon }\left({\text{x}}_{0}\right)=\underset{n}{\text{max }}{\dot{\varepsilon }}_{n}\left({\text{x}}_{0}\right)-\underset{n}{\text{min }}{\dot{\varepsilon }}_{n}\left({\text{x}}_{0}\right) \end{array}$$

Note that ${\dot{\varepsilon }}_{n}\left({\text{x}}_{0}\right)$ generally will be positive during inflation of a specified lung region, and negative during the corresponding deflation. Thus, $\Delta \dot{\varepsilon }\left({\text{x}}_{0}\right)$ will reflect the sum of the fastest inflation and deflation rates of the region.

Voxels within 6.0 mm of the image boundaries along the rostral-caudal axis were excluded from the mask for the purposes of image analysis, due to potential registration artifacts caused by motion of lung tissue into or out of the axial field of view. Regional aeration (Δ*I*), strain (Δε), and strain rate ($\Delta \dot{\varepsilon }$) were compared across subjects, lung conditions, and ventilation modalities by their mean value throughout the lung mask, coefficient of variation, and spatial gradients in the right-left, dorsal-ventral, and rostral-caudal directions. Spatial gradients for each variable were determined from the slopes computed by linear regression with respect to position along each of the principal anatomic axes. Spatial gradients were examined unnormalized and normalized by the spatial mean, in each case comparing both signed values of the gradient (i.e., positive and negative) and gradient magnitude (i.e., absolute value).

Given the harmonic nature of MFOV, the individual frequency components of *I*_*n*_, ε_*n*_, and ${\dot{\varepsilon }}_{n}$ were assessed using the Discrete Fourier Transform (DFT), to relate these periodic time-varying properties at each spatial position into harmonic amplitude and phase components. The regional heterogeneity of volumetric strain (Δε) was assessed using octree and supervoxel decomposition, each of which recursively subdivide regions-of-interest (ROIs) within the lung mask. Both decomposition methods were initialized with a single ROI containing the entire lung mask. At each recursive step, the designated ROI was subdivided into multiple new ROIs if the standard deviation of the contained voxel intensity was greater than a fixed threshold, set to a standard deviation of mean-normalized Δε ≥ 0.3.^2^ Octree decomposition, a three-dimensional extension of quadtree decomposition, was used to partition each ROI into eight octants according to bisecting coronal, sagittal, and transverse planes (Perchiazzi et al., 2014). Supervoxel decomposition was used to partition each ROI into two new ROIs by a weighted *k*-means clustering of voxels according to intensity similarity and spatial proximity (Conze et al., 2017). Recursive subdivision was continued until all ROIs either contained below-threshold values of standard deviation, or were <0.2% of the lung mask. Regional heterogeneity of Δε was then quantified by the mean ROI volume (${\bar{V}}_{\text{ROI}}$) as a fraction of the lung mask.

### Statistics

Non-parametric Kruskal-Wallis rank sum tests were performed for each outcome, testing for main effects of lung condition (i.e., baseline, injured) and ventilation modality (i.e., CMV, HFOV, MFOV) at the 0.05 significance level. The effect of ventilation modality was tested separately under baseline and injured conditions. For outcomes with a significant main effect of ventilation modality, differences among modalities were identified by a Dunn *post hoc* comparison with adjustment for rank ties and further adjustment by the Benjamini-Hochberg procedure for reduced false discovery rate. A web-based tool was used for statistical calculations (Vasavada, 2016).

## Results

**Figure 4:**
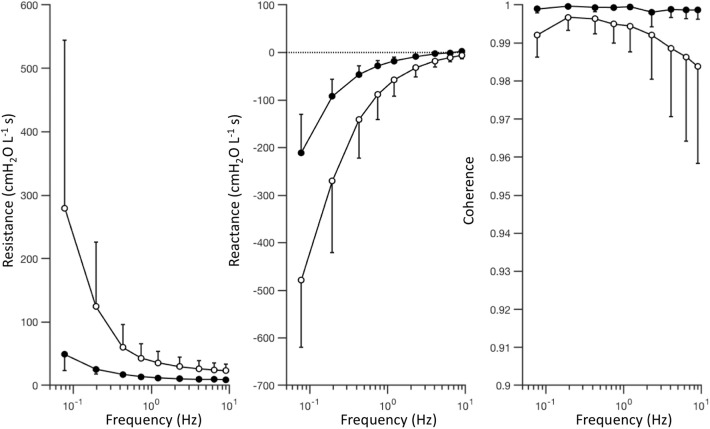
Respiratory impedance measured under baseline (black) and injured (white) conditions, represented by the mean value (circles) and standard deviation (error bars) at each frequency, across subjects. The complex-valued impedance spectrum is represented by its real part or in-phase component (i.e., resistance), and its imaginary part or out-of-phase component (i.e., reactance). Coherence provides an estimate of signal quality and linearity of the measured relationship between pressure and flow oscillations.

Figure 4 shows a summary of the respiratory impedance spectra *Z*_rs_ across all subjects measured under baseline and injured conditions, while Table 1 provides a summary of the constant phase model applied to *Z*_rs_. Under baseline conditions, average model-based tissue elastance *H* was 121 cmH_2_O L^−1^ at 1 rad s^−1^ (0.16 Hz) with a coefficient of variation of 0.31. The increase in *H* after oleic acid injury was highly variable, resulting in a mean of 435 cmH_2_O L^−1^ at 1 rad s^−1^ with a coefficient of variation of 0.94. By comparison, measurement of dynamic elastance (*E*_rs_) during CMV (Equation 5) was 148.9 ± 26.9 cmH_2_O L^−1^ and 257.5 ± 64.2 cmH_2_O L^−1^ under baseline and injured conditions, respectively. Resonant frequency (*f*_res_) increased after lung injury, in some cases above the measurement range allowed by the sampling parameters. Table 2 contains a summary of all significant effects identified by non-parametric Kruskal-Wallis tests, as well as *post hoc* comparisons as appropriate. These results are described further in the context of specific figures below.

**Table 1:** Respiratory system mechanics.

**Condition**	**${\bar{P}}_{aw}$^§^**	***R***	***I*_*aw*_**	**η**	***H***	***f*_*res*_^†^**	***E*_*rs*_^‡^**
Baseline	11.5 ± 0.8	8.9 ± 3.2	0.11 ± 0.03	0.17 ± 9.93	121 ± 38	6.7 ± 1.7	149 ± 27
Injured	12.6 ± 2.9	22.4 ± 13.5	0.13 ± 0.08	0.27 ± 0.04	435 ± 407	9.2 ± 1.3	258 ± 64

§ *${\bar{P}}_{aw}$ during respiratory impedance measurements*.

† *f_res_ interpolation was possible only for nine subjects under baseline conditions and five subjects under injured conditions, due to resonance in other cases occurring above the 12 Hz low-pass filter cutoff frequency*.

‡ *E_rs_ measurements obtained during conventional mechanical ventilation by Equation (5). ${\bar{P}}_{aw}$, mean airway pressure (cmH_2_O); R, Newtonian airway and chest wall resistance (cmH_2_O L^-1^ s); I_aw_, airway inertance (cmH_2_O L^-1^ s^2^); η, tissue hysteresivity; H, tissue elastance (cmH_2_O L^-1^ at 1 rad s^-1^); f_res_, resonant frequency (Hz); E_rs_, dynamic respiratory system elastance (cmH_2_O L^−1^)*.

**Table 2:**
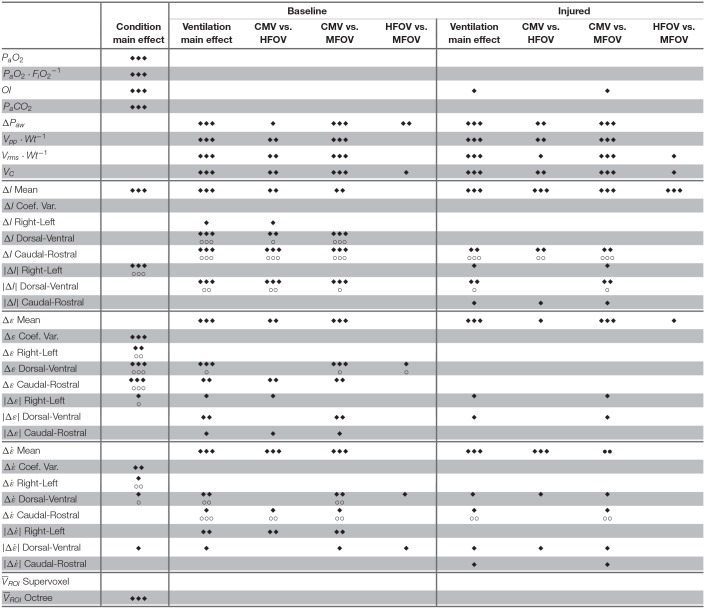
Statistically significant effects determined by Kruskal-Wallis rank sum test for main effects of lung condition (i.e., baseline, injured) and ventilation modality (i.e., CMV, HFOV, MFOV).

*Post-hoc comparisons for ventilation modality performed using the Dunn method with Benjamini-Hochberg adjustment. (◆p < 0.05, ◆◆p < 0.01, ◆◆◆p < 0.001). Symbols distinguish unnormalized (◆) and mean-normalized (°) spatial gradients. P_a_O_2_, arterial oxygen tension; F_i_O_2_, fractional inspired oxygen; OI, oxygenation index; ΔP_aw_, pressure amplitude; V_pp_, peak-peak volume; Wt, body weight; V_rms_, root-mean-square volume; V_C_, ventilatory cost function; P_a_CO_2_, arterial carbon dioxide tension; ΔI, range of CT value; Δε, range of volumetric strain; $\Delta \dot{\varepsilon }$, range of volumetric strain rate; ${\bar{V}}_{ROI}$, mean cluster volume; |⋯|, absolute value of enclosed argument*.

**Figure 5:**
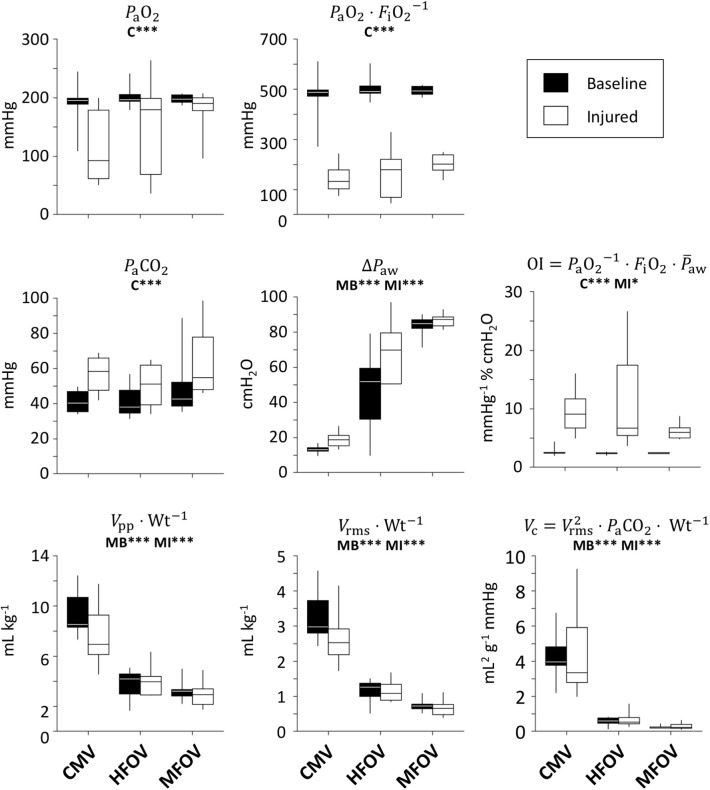
Gas exchange outcomes, represented by box plots showing minimum, maximum, and quartiles across subjects under baseline (black) and injured (white) conditions, during conventional mechanical ventilation (CMV), high-frequency oscillatory ventilation (HFOV), and multi-frequency oscillatory ventilation (MFOV). Asterisks indicate significance levels (*p < 0.05, ***p < 0.001) for main effects (C = lung condition, MB = modality for baseline condition, MI = modality for injured condition). See Table 2 for additional clarification. *P*_a_O_2_ = arterial oxygen tension; *F*_i_O_2_ = fractional inspired oxygen; *P*_a_CO_2_ = arterial carbon dioxide tension; Δ*P*_aw_ = airway pressure amplitude; *V*_pp_ = peak-to-peak volume range; Wt = body weight; *V*_rms_ = root-mean-square volume.

Figure 5 shows gas exchange outcomes related to mechanical ventilation, including oxygenation index ($\text{OI}={P_{\text{a}}{\text{O}}_{2}}^{-1}\cdot F_{\text{i}}{\text{O}}_{2}\cdot {\bar{P}}_{\text{aw}}$) and ventilatory cost function ($V_{\text{C}}=P_{\text{a}}{\text{CO}}_{2}\cdot V_{\text{rms}}^{2}\cdot {\text{Wt}}^{-1}$). A significant main effect of lung condition (baseline vs. injured) was found for *P*_a_O_2_, $P_{\text{a}}{\text{O}}_{2}\cdot {F_{\text{i}}{\text{O}}_{2}}^{-1}$, OI, and *P*_a_CO_2_ (*p* < 0.001). A significant main effect of ventilation modality was found in Δ*P*_aw_, $V_{\text{pp}}\cdot {\text{Wt}}^{-1}$, $V_{\text{rms}}\cdot {\text{Wt}}^{-1}$, and *V*_C_ (*p* < 0.001) for both lung conditions, as well as in OI for the injured condition (*p* < 0.05). In particular, median OI increased by 300% for injured subjects compared to baseline (*p* < 0.001), but was 48% lower for injured subjects during MFOV compared to CMV (*p* < 0.05). MFOV and HFOV were associated with lower median *V*_*C*_ for both baseline and injured conditions compared to CMV (HFOV 84% lower than CMV, *p* < 0.01; MFOV 93% lower than CMV, *p* < 0.001). Pairwise comparison of *V*_*C*_ for MFOV against HFOV also achieved a statistically significant difference, with 60% lower median *V*_C_ for MFOV compared to HFOV (*p* < 0.05). Median $V_{\text{rms}}\cdot {\text{Wt}}^{-1}$ was 41% lower for MFOV vs. HFOV (*p* = 0.0505 baseline; *p* < 0.05 injured) and 75% lower for MFOV vs. CMV (*p* < 0.001).

**Figure 6:**
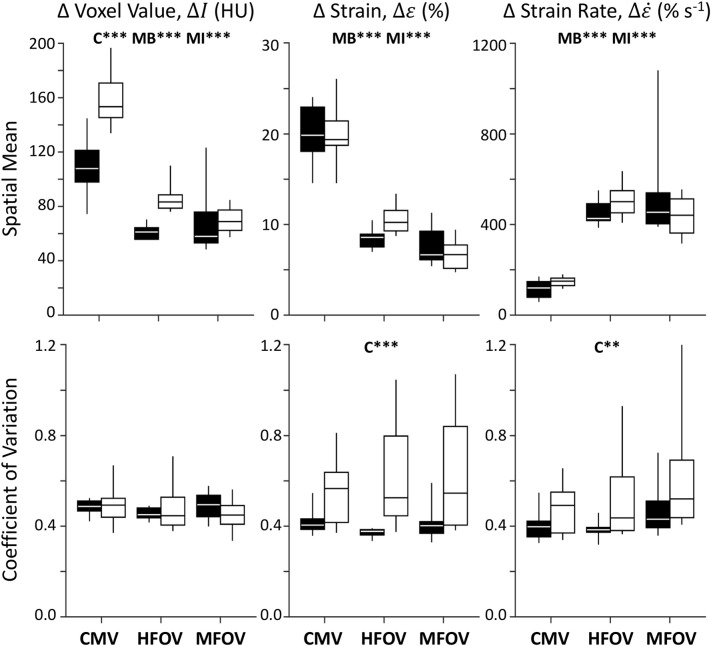
Summary of image registration-based regional ventilation measures for intratidal changes in aeration (Δ*I*), volumetric strain (Δε), and volumetric strain rate ($\Delta \dot{\varepsilon }$). Mean and coefficient of variation throughout the lung mask are shown for each variable, represented by box plots showing minimum, maximum, and quartiles across subjects under baseline (black) and injured (white) conditions, during conventional mechanical ventilation (CMV), high-frequency oscillatory ventilation (HFOV), and multi-frequency oscillatory ventilation (MFOV). Asterisks indicate significance levels (**p < 0.01, ***p < 0.001) for main effects (C = lung condition, MB = modality for baseline condition, MI = modality for injured condition). See Table 2 for additional clarification.

Figure 6 shows registration-based estimates of regional ventilation, as intratidal changes in voxel aeration (Δ*I*), volumetric strain (Δε), and volumetric strain rate ($\Delta \dot{\varepsilon })$. A significant main effect of condition (baseline vs. injured) was found for Δ*I* spatial mean (*p* < 0.001), Δε coefficient of variation (*p* < 0.001) and $\Delta \dot{\varepsilon }$ coefficient of variation (*p* < 0.01). A significant main effect of ventilation modality was found for the spatial means of all three variables—Δ*I*, Δε, and $\Delta \dot{\varepsilon }$–in both baseline and injured lung conditions (*p* < 0.001). In particular, MFOV and HFOV both produced smaller Δ*I*, smaller Δε, and larger $\Delta \dot{\varepsilon }$ compared to CMV under baseline and injured conditions (*p* < 0.05). For the injured condition, MFOV resulted in significantly lower Δ*I* and Δε compared to HFOV (*p* < 0.05), but no difference for $\Delta \dot{\varepsilon }$ (*p* = 0.20).

**Figure 7:**
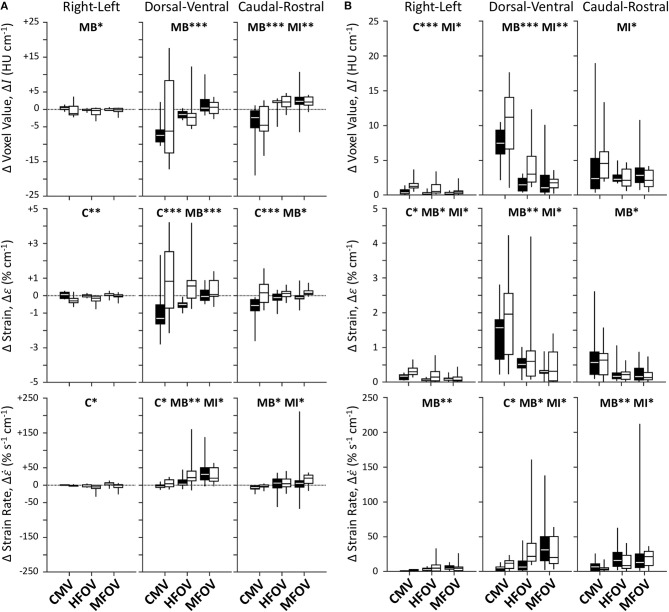
Unnormalized spatial gradient values **(A)** and magnitudes **(B)** for image registration-based regional ventilation measures of intratidal changes in aeration (Δ*I*), volumetric strain (Δε), and volumetric strain rate ($\Delta \dot{\varepsilon }$). Linear spatial gradients in each of the three principal anatomic directions throughout the lung mask are shown for each variable, represented by box plots showing minimum, maximum, and quartiles across subjects under baseline (black) and injured (white) conditions, during conventional mechanical ventilation (CMV), high-frequency oscillatory ventilation (HFOV), and multi-frequency oscillatory ventilation (MFOV). Asterisks indicate significance levels (*p < 0.05, **p < 0.01, ***p < 0.001) for main effects (C = lung condition, MB = modality for baseline condition, MI = modality for injured condition). See Table 2 for additional clarification.

**Figure 8:**
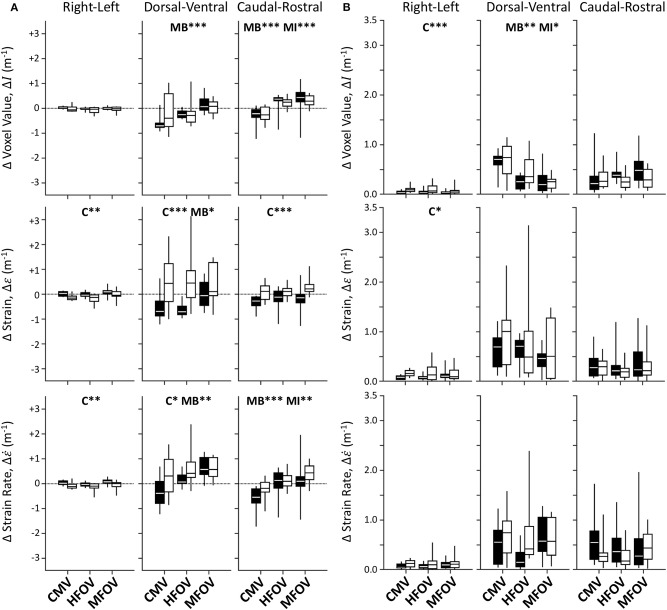
Mean-normalized spatial gradient values **(A)** and magnitudes **(B)** for image registration-based regional ventilation measures of intratidal changes in aeration (Δ*I*), volumetric strain (Δε), and volumetric strain rate ($\Delta \dot{\varepsilon }$). Linear spatial gradients in each of the three principal anatomic directions throughout the lung mask are shown for each variable, represented by box plots showing minimum, maximum, and quartiles across subjects under baseline (black) and injured (white) conditions, during conventional mechanical ventilation (CMV), high-frequency oscillatory ventilation (HFOV), and multi-frequency oscillatory ventilation (MFOV). Asterisks indicate significance levels (*p < 0.05, **p < 0.01, ***p < 0.001) for main effects (C = lung condition, MB = modality for baseline condition, MI = modality for injured condition). See Table 2 for additional clarification.

Figure 7 shows unnormalized spatial gradients of Δ*I*, Δε, and $\Delta \dot{\varepsilon }$ in the right-left, dorsal-ventral, and caudal-rostral axes. The largest spatial gradient magnitudes observed occurred along the dorsal-ventral axis. In particular, the injured lung condition was associated with significantly more positive dorsal-ventral gradients of Δε (*p* < 0.001) and $\Delta \dot{\varepsilon }$ (*p* < 0.05). Ventilation modality had a significant effect on all dorsal-ventral gradients under baseline conditions (Δ*I*, *p* < 0.001; Δε, *p* < 0.001; $\Delta \dot{\varepsilon }$, *p* < 0.01), with MFOV producing the least negative dorsal-ventral gradients of Δ*I* and Δε, but the most positive dorsal-ventral gradients of $\Delta \dot{\varepsilon }$. Although modality was not a significant predictor of variability in the actual value (positive or negative) of any dorsal-ventral gradient for injured subjects, it was a significant main effect for the *magnitudes* of all dorsal-ventral gradients (Δ*I*, *p* < 0.001; Δε, *p* < 0.01; $\Delta \dot{\varepsilon }$, *p* < 0.05). In general HFOV and MFOV reduced the dorsal-ventral gradient magnitudes of Δ*I* and Δε under both baseline and injured conditions, while increasing the corresponding $\Delta \dot{\varepsilon }$ gradient. Figure 8 shows corresponding mean-normalized spatial gradients. In general, normalizing each spatial gradients by the respective spatial mean accounted for much of the variability observed in the unnormalized spatial gradients (Figure 7) with respect to varying ventilation modality. This is also evidenced by the loss of significant differences between ventilation modalities for the corresponding spatial gradients as indicated in Table 2.

**Figure 9:**
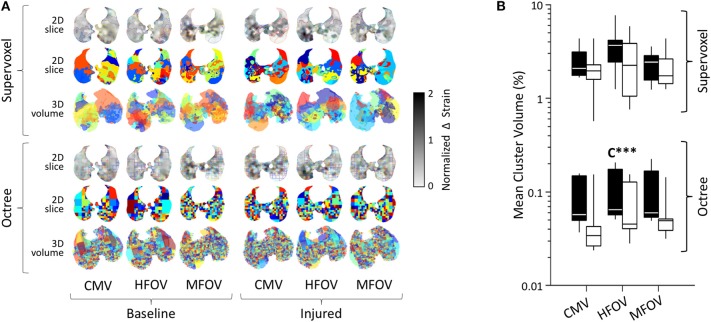
Regional patchiness of intratidal variations in volumetric strain (Δε) during conventional mechanical ventilation (CMV), high-frequency oscillatory ventilation (HFOV), and multi-frequency oscillatory ventilation (MFOV). Regional patchiness was assessed by the size, number, and distribution of regions-of-interest or clusters (randomized colors) obtained by recursive supervoxel or octree decomposition. Examples from a representative subject **(A)**, and cluster volumes as a fraction of the lung mask **(B)** represented by box plots showing minimum, maximum, and quartiles across subjects under baseline (black) and injured (white) conditions. Asterisks indicate significance levels (***p < 0.001) for main effects (C = lung condition).

Figure 9 shows an example of regions-of-interest defined by recursive supervoxel and octree decomposition performed on mean-normalized Δε in one subject. Clustered regions are illustrated using contours and filled regions in two-dimensional views of a slice, as well as translucent surface renderings in three-dimensional projections. Note that supervoxel decomposition produces larger contiguous ROIs, despite using the same decision criteria for recursive cluster subdivision. Figure 9 also provides a summary of mean cluster size (${\bar{V}}_{\text{ROI}}$) across subjects, condition, and ventilation modality. A significant effect of lung condition was found for the octree technique (*p* < 0.001), with 32% smaller median ${\bar{V}}_{\text{ROI}}$ in injured lungs.

**Figure 10:**
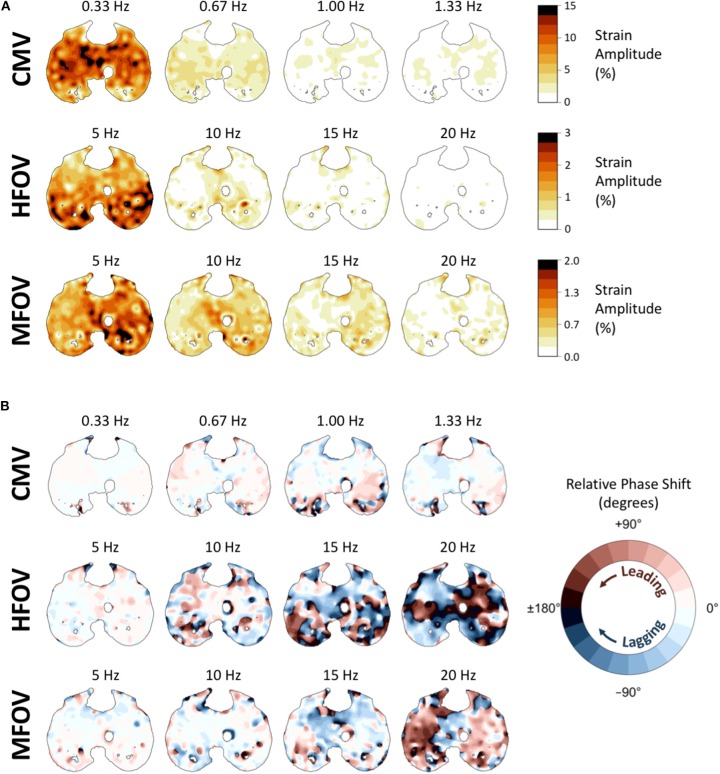
Frequency-domain representations of tissue-correlated regional strain amplitude **(A)** and phase variations **(B)** in one subject during conventional mechanical ventilation (CMV), high-frequency oscillatory ventilation (HFOV), and multi-frequency oscillatory ventilation (MFOV). For each ventilation modality, strain amplitudes and phases were obtained from the discrete Fourier transform of time-varying Jacobian determinants within each voxel, using transformations mapped to a single reference image. In each cross-section of the reference image, voxel color in **(A)** indicates the strain amplitude at that frequency, corresponding to the amplitude of volumetric strain relative to the minimum volume of that particular voxel. Voxel color in **(B)** indicates the amount of phase shift (leading or lagging) relative to the median phase of all lung voxels at the specified frequency.

**Figure 11:**
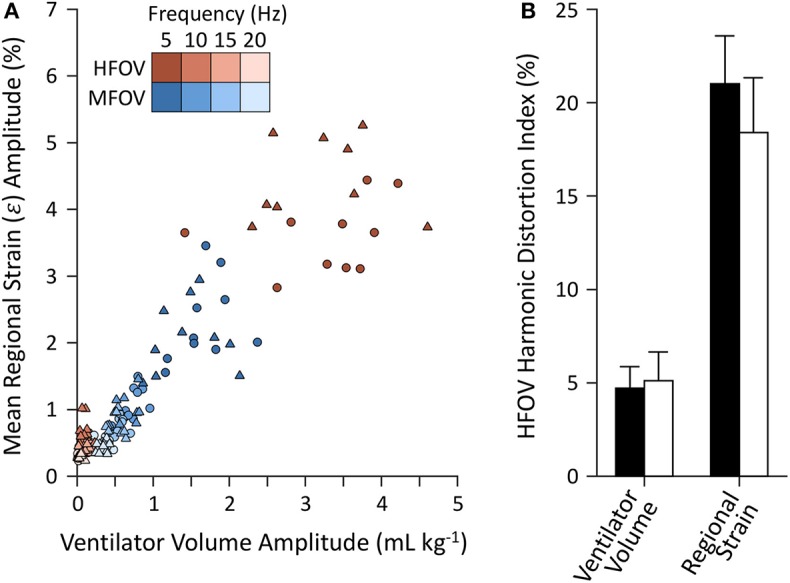
Harmonic amplitudes **(A)** from imaging-based regional strain measurements and corresponding amplitudes from the ventilator volume waveform measured at the airway opening under baseline (circles) and injured (triangles) conditions. Harmonic distortion index **(B)** mean and standard deviation for each set of measurements, grouped by baseline (black) and injured (white) lung conditions.

Figure 10 shows example distributions of strain amplitude and phase at the first four harmonics for CMV, HFOV, or MFOV, computed using the discrete Fourier transform of time-varying Jacobian determinants in aligned images. Figure 11 provides a summary of the harmonic strain amplitudes during HFOV and MFOV across all subjects, correlated with corresponding amplitudes of the ventilator volume waveform *V*(*t*). The oscillatory waveforms were a 5 Hz sinusoid (HFOV) and a combination of 5, 10, 15, and 20 Hz sinusoids with uniform flow amplitudes (MFOV). The relative amplitude distributions in the flow waveforms delivered by the ventilator were largely preserved throughout the lung, according to the registration-based metrics (*r*^2^ = 0.78). However, harmonic distortion measurements during HFOV indicated nearly 20% of the spectral power in regional strain was concentrated in the higher-order harmonics of the 5 Hz waveform (i.e., 10, 15, 20 Hz), whereas the ventilator volume waveform exhibited only 5% harmonic distortion (Zhang et al., 1995; Amini et al., 2017).

## Discussion

In this study, we demonstrated that oscillatory ventilation improves the average regional lung strain, as well as the spatial gradients of lung strain, compared to a conventional pressure-controlled modality in pigs with heterogeneous lung injury. This study revealed a mechanism by which enhanced harmonic content in MFOV waveforms may improve gas exchange compared to either CMV or single-frequency HFOV waveforms.

The increase in respiratory system elastance following lung injury (i.e., 183% increase in *E*_rs_ and 355% increase in *H*) is consistent with substantial lung derecruitment and/or surfactant dysfunction. Consequently, increased driving pressure was required to maintain eucapnia at comparable respiratory rates. Despite similar $V_{\text{pp}}\cdot {\text{Wt}}^{-1}$ and ventilatory cost function (*V*_C_), the driving pressure (Δ*P*_aw_) after lung injury increased to 18.7 cmH_2_O, compared to 13.0 cmH_2_O at baseline (Figure 5). Increased Δ*P*_aw_ during CMV after injury exceeded the 15 cmH_2_O threshold identified by Amato et al. (2015), above which the mortality odds ratio was greater than unity in patients with ARDS. The use of Δ*P*_aw_ > 15 cmH_2_O suggests substantial potential for VILI despite average measured $V_{\text{pp}}\cdot {\text{Wt}}^{-1}$ of 7.5 mL kg^−1^ (Figure 5). However, such heuristics are only applicable during CMV, for which *P*_aw_ fluctuations are well-correlated with lung strain. During oscillatory modes of ventilation, Δ*P*_aw_ as computed from Equation (6) does not represent elastic pressures distending peripheral alveoli, but also includes substantial pressure losses due to resistive and inertial loads imposed by the endotracheal tube and conducting airways, especially with increasing frequency. The fact that Δ*P*_aw_ was greater during MFOV than HFOV indicates that larger pressure amplitudes were required to overcome the increased resistive and inertial losses at frequencies up to 20 Hz compared to 5 Hz, but does not indicate that these larger airway pressures were transmitted to distal alveoli (Pillow et al., 2002). We found that the spatial mean of strain throughout the entire lung during CMV was unchanged after injury, despite increased Δ*P*_aw_ and Δ*I* spatial mean (Figure 6). Some evidence to reconcile this discrepancy is apparent in the greatly increased coefficient of variation for Δε (Figure 6). Upon closer examination of the regional strain distributions in injured lungs, Δε was nearly zero in consolidated regions of the lung with *I* >-100 HU. By contrast, Δε was increased by 25% in normally aerated regions (i.e., −900 HU < *I* < −500 HU) relative to the average throughout the entire lung mask. Thus, the increased Δ*P*_aw_ required to deliver similar $V_{\text{pp}}\cdot {\text{Wt}}^{-1}$ (Figure 5) resulted in concentrated strains within the recruited lung. This finding is consistent with the concept of the “baby lung,” which describes reduced functional recruited volume in ARDS (Gattinoni and Pesenti, 2005; Gattinoni et al., 2016a).

Spatial gradients of intratidal strain also provide some insight into the redistribution of mechanical flows after lung injury. As expected, the magnitudes of right-left gradients were small relative to those of the dorsal-ventral and caudal-rostral gradients (Figure 7). Most noticeably, lung injury was associated with dramatic changes in dorsal-ventral Δε gradients. Ventilation in healthy subjects during CMV tended to distribute toward the dorsal and caudal lung regions. However, varying degrees of lung injury resulted in consolidation or derecruitment of the dependent lung, such that dorsal-ventral Δε gradients were reduced (or even reversed) depending on the extent of edema, particularly for CMV and HFOV. Interestingly, intratidal changes in aeration tended to preserve the dorsal-ventral Δ*I* gradient before and after injury, despite the positive shift observed in dorsal-ventral Δε gradients. It should be noted that actual spatial distributions of Δ*I* and Δε are likely not well-described by linear functions of spatial position following lung injury, and may even be non-monotonic (Johnson et al., 2017). For example, negative dorsal-ventral gradients may be possible in the recruited lung, but counteracted by zero ventilation in the derecruited dependent lung such that the resulting overall linear gradient may be negative, zero, or even positive. Nonetheless compared to either CMV of HFOV, MFOV reduced the magnitude of spatial gradients in ventilation regardless of the lung condition. Other strategies for improving ventilation distribution and eliminating large dorsal-ventral gradients include prone position ventilation (Scholten et al., 2017; Xin et al., 2018), which was not used in this study. Prone posture serves to eliminate large-scale gravitational gradients in lung expansion (Hoffman, 1985), but would not be expected to affect the local, region-to-region mechanical differences which MFOV targets. Finally, both HFOV and MFOV were associated with more positive caudal-rostral Δ*I* gradients compared to CMV, regardless of injured condition (*p* < 0.001). CMV-associated findings indicate that in contrast to oscillatory modalities, CMV preferentially ventilates the basal regions of the lungs, consistent with a previous positron emission tomography study (Venegas et al., 1993).

Much of the variability in spatial gradient magnitudes across different ventilation modalities was due to changes in the spatial means. By contrast, the normalized gradients (i.e., the spatial gradients divided by the spatial mean) exhibited fewer significant differences across modalities (Figure 8, Table 2). This finding indicates that, at least for the particular frequencies and waveforms used in this study, the *relative* spatial gradients were not substantially altered by modality. This may be due in part to the predominance of the 5 Hz component in both HFOV and MFOV waveforms, which was below the resonant frequency in almost every case (Table 1), and therefore below a threshold frequency for developing regional ventilation heterogeneity due to resonant amplification (Herrmann et al., 2019a). Our results then indicate that broadband MFOV waveforms, while producing the same *relative* ventilation heterogeneity as HFOV, still reduce the average strain and magnitude of strain gradients that are associated with risk for VILI.

Based on our supervoxel and octree analysis, regional strain heterogeneity did not differ greatly across ventilation modality, although the injured lungs did require smaller sizes to describe the spatial clustering of Δε compared to the baseline conditions (Figure 9). Lung injury may thus result in increased small-scale strain heterogeneity (Kaczka et al., 2011; Perchiazzi et al., 2014). It should be noted that our estimates of regional strain derived from image registration are inherently smooth due to the use of B-splines to define the spatiotemporal deformations. The discrepancy between the supervoxel- and octree-based results may also be due in part to the discrete anatomic structure of lungs, which consist of an assumed continuum of spongy parenchymal tissues penetrated by branching networks of airways and vessels. Airways and vessels are not expected to undergo large volumetric strains during mechanical ventilation, and therefore give rise to irregularities in the spatial field of Δε. Such irregularities may be approximately contoured by supervoxel boundaries, which are not rigidly constrained. By contrast, octree decomposition is strictly planar, and therefore may require greater degrees of recursive subdivision to isolate such irregular spatial distributions of Δε (Perchiazzi et al., 2014). The supervoxel decomposition incorporates spatial proximity as well as image intensity, such that reducing the spatial proximity weighting may facilitate the identification of larger clusters albeit with irregular boundaries. Therefore, it is possible that our findings would be altered if smaller airways and blood vessels were further segmented from the lung parenchyma prior to cluster analysis, or if a different spatial proximity weighting was used for the supervoxel decomposition.

Separation of the harmonic content of regional ventilation illustrates a potential influence of nonlinear mechanics during HFOV at 5 Hz (Figure 11). For example, image registration resulted in estimation of $\dot{\varepsilon }$ amplitudes at 10, 15, and 20 Hz of roughly one third those at 5 Hz, despite the ventilator waveform consisting of only 5 Hz oscillation (Supplementary Figure S-4). By contrast, $\dot{\varepsilon }$ amplitudes during MFOV approximated the input distribution of uniform amplitude flow at all four harmonics (Supplementary Figure S-4). Intrapulmonary harmonic distortion of distributed flows during HFOV (with only a single frequency) may result from the use of larger-amplitude flows exacerbating inherent mechanical nonlinearities, but may also result from the complex interaction of mechanically-interdependent lung tissues (Suki and Bates, 1991; Suki et al., 1991). During MFOV, the use of small-amplitude flows may contribute to relatively linear mechanical behavior of the lung tissues. It is also possible that the observed nonlinearity may be due in part to imaging artifact and errors in image registration resulting in misidentified high-frequency, small-amplitude motion. Assuming that our registration results are accurate however, the $\dot{\varepsilon }$ amplitudes measured at the higher harmonics during HFOV are surprisingly large, almost comparable to those intentionally delivered during MFOV. It is plausible that the mechanisms by which MFOV may enhance gas transport already occur, at least to some extent, within the lung periphery during HFOV. However, MFOV may induce these higher harmonics directly, without incurring the overhead of generating high flow rates at the fundamental frequency. The consequences of such overhead may include, for example, increased spatial gradients of strain and strain rate due to gravitational dependence (Figure 7).

### Limitations

MFOV may improve ventilation distribution and the efficiency gas exchange efficacy (Kaczka et al., 2015; Herrmann et al., 2018, 2019b). However, given the technical limitations of the mechanical ventilator as well as our dynamic CT image reconstruction technique which assumed strict periodicity of motion (Herrmann et al., 2017), the MFOV waveform used in this study comprised only harmonic components of the fundamental frequency. Cardiogenic motion was not synchronized with respiratory motion and therefore presents a source of artifact in the reconstructed 4DCT images of respiratory motion, depending on the relative periodicity between the ventilator frequency and the heart rate. Such artifact may manifest as rapid oscillations in CT density (Herrmann et al., 2017), which may influence registration-based estimates of strain rate, especially in regions adjacent to the mediastinum.

In addition, the interpretation of regional strain in the proximity of recruitment/derecruitment is challenging for several reasons. Standard image registration techniques (e.g., using a similarity cost function defined as the sum of squared intensity differences) assume a uniform intensity transform to predict voxel-wise correspondence, but cannot account for local changes in intensity associated with changing gas fraction. Consequently, these approaches may result in the prediction of non-physical deformations, such as contraction of nonaerated lung regions. The sum of squared tissue volume differences (SSTVD) similarity cost function used in this study addresses this problem using a non-uniform intensity transform that accounts for local variations in CT intensity that are due to changing proportions of gas-to-tissue content in each lung region during breathing or ventilation (Zhao et al., 2016). Another challenge to registration of injured lungs is the lack of contrast or structural landmarks in consolidated regions, which renders validation of estimated voxel-wise correspondence nearly impossible, especially when large changes in recruitment occur between two images. In this study, high temporal resolution imaging (7–105 Hz) was used to minimize the time between adjacent ventilation phases, which resulted in small deformations and small changes in recruitment between adjacent images being registered. Linear elastic regularization produces plausible predictions of voxel-wise correspondence in regions with little contrast, assuming the lung parenchyma deforms as a linear elastic material. An important limitation of most registration techniques is the assumption of smoothly varying deformation, which may not accurately reflect discontinuous motion at distinct structural boundaries between aerated and nonaerated lung tissue. Due to the limited spatial resolution in this study (0.6 mm), each voxel may contain up to dozens of alveoli. Thus, it is difficult, if not impossible, to distinguish the underlying cause of observed changes in aeration (Cereda et al., 2019). For example, the same change in aeration of a single voxel may represent uniform deflation of its alveoli, complete derecruitment of only a portion of alveoli, or any combination thereof. Thus, prediction of recruitment/derecruitment on the basis of voxel aeration is speculative.

Finally, while our study focused on regional distributions of aeration, strain, and strain rate as the primary indicators of ventilation and VILI, the exact relationship between these mechanical variables and VILI is difficult to ascertain. VILI is a complicated process associated with a diverse range of mechanical stimuli (Hussein et al., 2013; Carrasco Loza et al., 2015; Güldner et al., 2016; Smith et al., 2017; Tonetti et al., 2017). So-called “mechanical power” (i.e., the rate of energy dissipation across parenchymal tissues) may provide a better prediction of VILI compared to either strain or strain rate alone (Gattinoni et al., 2016b; Serpa Neto et al., 2018). However, measurement of regional mechanical power is not practical, given the requirement of regional gas pressure within the lung for its estimation. Alternatively, VILI may also be assessed using fluoro-deoxyglucose (FDG) uptake (Wellman et al., 2014), serum or alveolar inflammatory cytokines (Liu et al., 2013), protein content in bronchoalveolar lavage fluid (Smith et al., 2017), and histopathology. All of these quantitative measurements of lung injury require extensive durations of mechanical ventilation prior to measurement, some of which may only be obtained *post mortem*.

### Conclusion

In an oleic acid model of porcine ARDS, high-frequency and multi-frequency oscillatory ventilation resulted in improved gas exchange efficiency and reduced regional lung strain compared to conventional mechanical ventilation. MFOV also reduced spatial gradients in lung strain compared to CMV or HFOV, resulting in a more uniform overall distribution of lung strain. By contrast, mean-normalized spatial gradients exhibited less dependence on ventilation modality, indicating that the use of higher harmonic frequencies in MFOV does not substantially alter *relative* ventilation heterogeneity. Thus, the reduced spatial strain gradients observed with MFOV, compared to HFOV or CMV, may be explained by reductions in the *average* parenchymal strain. In summary, MFOV supports gas exchange with reduced lung strain, and may provide additional benefit over HFOV by reducing ventilation heterogeneity associated with spatial gradients in absolute strain, and by reducing the strain associated with conventional ventilation or single-frequency oscillation.

## Data Availability Statement

The datasets generated for this study are available on request to the corresponding author.

## Ethics Statement

The animal study was reviewed and approved by University of Iowa Institute for Animal Care and Use Committee (Protocol Number 5061428).

## Author Contributions

JH and DK conceived the study, collected the data, and wrote the manuscript. JH, SG, WS, MH, JR, GC, EH, and DK analyzed the data and revised the manuscript.

### Conflict of Interest

JH and DK are co-founders and shareholders of OscillaVent, Inc. JR and EH are co-founders and shareholders of VIDA Diagnostics, Inc. The remaining authors declare that the research was conducted in the absence of any commercial or financial relationships that could be construed as a potential conflict of interest.

## Figures and Tables

**Table:** 

4DCT	Four-dimensional computed tomography
ARDS	Acute respiratory distress syndrome
CMV	Conventional mechanical ventilation
CO_2_	Carbon dioxide
*E*_rs_	Dynamic respiratory system elastance
FDG	Fluorodeoxyglucose
*f*	Frequency
*f*_0_	Fundamental frequency
*f*_res_	Resonant frequency
*F*_i_O_2_	Fraction of oxygen in inspired gas
HFOV	High-frequency oscillatory ventilation
*H*	Tissue elastance
*I*_aw_	Airway inertance
*I*	Volumetric CT density image
*I*_avg_	Target image for registration, representing the average of deformed images
*I*_*n*→*m*_	Result of deforming the*n*th image into the*m*th spatial reference frame
Δ*I*	Intratidal range of CT density
$J_{n\to m}$	Jacobian matrix of the transformation from the *n*th to *m*th spatial reference frames
*j*	Unit imaginary number, i.e., $j=\sqrt{-1}$
*k*	Frequency index
MFOV	Multi-frequency oscillatory ventilation
*M*	Lung segmentation mask
*m*	Image index, corresponding to phase of periodic ventilation cycle
*n*	Image index, corresponding to phase of periodic ventilation cycle
*N*	Number of images in periodic sequence
OI	Oxygenation index
*P*	Pressure
*P*_aw_	Pressure at the airway opening
${\bar{P}}_{\text{aw}}$	Mean airway pressure
Δ*P*_aw_	Intratidal range of airway pressure
*P*_a_CO_2_	Partial pressure of carbon dioxide in arterial blood
*P*_a_O_2_	Partial pressure of oxygen in arterial blood
PEEP	Positive end-expiratory pressure
ROI	Region of interest
*R*	Airway resistance
SFOV	Single-frequency oscillatory ventilation
SSTVD	Sum of squared tissue volume differences
*t*	Time
δ*t*	Time step between adjacent image phases
*T*	Time duration of the ventilatory cycle
VILI	Ventilator-induced lung injury
*V*	Volume
$\bar{V}$	Time-averaged volume over the ventilatory cycle
*V*_pp_	Peak-to-peak volume
*V*_rms_	Root-mean-square volume
${\bar{V}}_{\text{ROI}}$	Mean cluster size after recursive octree or supervoxel decomposition
δ*V*	Volume of a single voxel
*V*_C_	Ventilatory cost function
Wt	Body weight
*x*_*n*_	Vector of 3-dimensional spatial coordinates in*n*th image reference frame
*X*	Vector of 3-dimensional spatial coordinates in target image reference frame
*X*_rs_	Respiratory system reactance
*Z*_rs_	Respiratory system mechanical impedance
Ẑ_rs_	Model-predicted respiratory system mechanical impedance
α	Exponent of frequency in constant phase model
ε	Volumetric strain
Δε	Intratidal range of volumetric strain
$\dot{\varepsilon }$	Volumetric strain rate
$\Delta \dot{\varepsilon }$	Intratidal range of volumetric strain rate
γ^2^	Coherence spectrum
η	Tissue hysteresivity
π	Number of radians in a semicircle
ϕ	4-dimensional transformation mapping image sequence to target average image
∇_*x*_	Spatial gradient operator

## Footnotes

^1^The use of *V*_rms_ enables comparison between single-frequency modalities, which are typically described by a single tidal volume, and MFOV, which is described by a distinct volume amplitude at each frequency (Kaczka et al., 2015).

^2^This threshold value was empirically chosen to be lower than the values of the regional strain coefficient of variation observed across all subjects, to ensure that the recursive subdivision would not be terminated trivially at the level of the entire lung mask, yet high enough to avoid termination at the level of individual voxels. The same threshold was used for all normalized images.
