# Supplementary material for: Quantifying Regional Lung Deformation Using Four-Dimensional Computed Tomography: A Comparison of Conventional and Oscillatory Ventilation
Source: Front Physiol. 2020 Feb 20;11:14. doi: 10.3389/fphys.2020.00014 (PMC7044245; doi:10.3389/fphys.2020.00014)
Supplement: Supplementary file 1 [file Table_1.DOCX]

Supplementary Material

# Supplementary Data

Supplementary Animation S-1. Example computed tomographic image sequence for a subject under baseline conditions during conventional mechanical ventilation. One axial slice at a fixed spatial position is shown over time (**Original CT**). After 4D image registration, the deformed images appear relatively motionless, indicating alignment of deformed respiratory structures with their configuration in the reference ventilation phase (**Deformed**). CT intensity in the deformed images are used to assess tissue-correlated aeration throughout the ventilatory cycle (**Aeration**), i.e., intratidal changes in the relative fraction of gas vs. water contained within a fixed region of interest with respect to the reference ventilation phase. The determinant of the deformation gradient is used to assess tissue-correlated volumetric strain (**Strain**), i.e., the relative increase in volume of a fixed region of interest with respect to volume at the reference ventilation phase. In this case, the reference ventilation phase was aligned with the end of expiration, such that the volumetric strain is 0% at end-expiration and increases to positive values (red) during inspiration.

Supplementary Animation S-2. Example computed tomographic image sequence for a subject under injured conditions during conventional mechanical ventilation. One axial slice at a fixed spatial position is shown over time (**Original CT**). After 4D image registration, the deformed images appear relatively motionless, indicating alignment of deformed respiratory structures with their configuration in the reference ventilation phase (**Deformed**). CT intensity in the deformed images are used to assess tissue-correlated aeration throughout the ventilatory cycle (**Aeration**), i.e., intratidal changes in the relative fraction of gas vs. water contained within a fixed region of interest with respect to the reference ventilation phase. The determinant of the deformation gradient is used to assess tissue-correlated volumetric strain (**Strain**), i.e., the relative increase in volume of a fixed region of interest with respect to volume at the reference ventilation phase. In this case, the reference ventilation phase was aligned with the end of expiration, such that the volumetric strain is 0% at end-expiration and increases to positive values (red) during inspiration. Note how regions that remain non-aerated throughout the entire ventilatory cycle also exhibit near-zero volumetric strain.

# Supplementary Figures

**
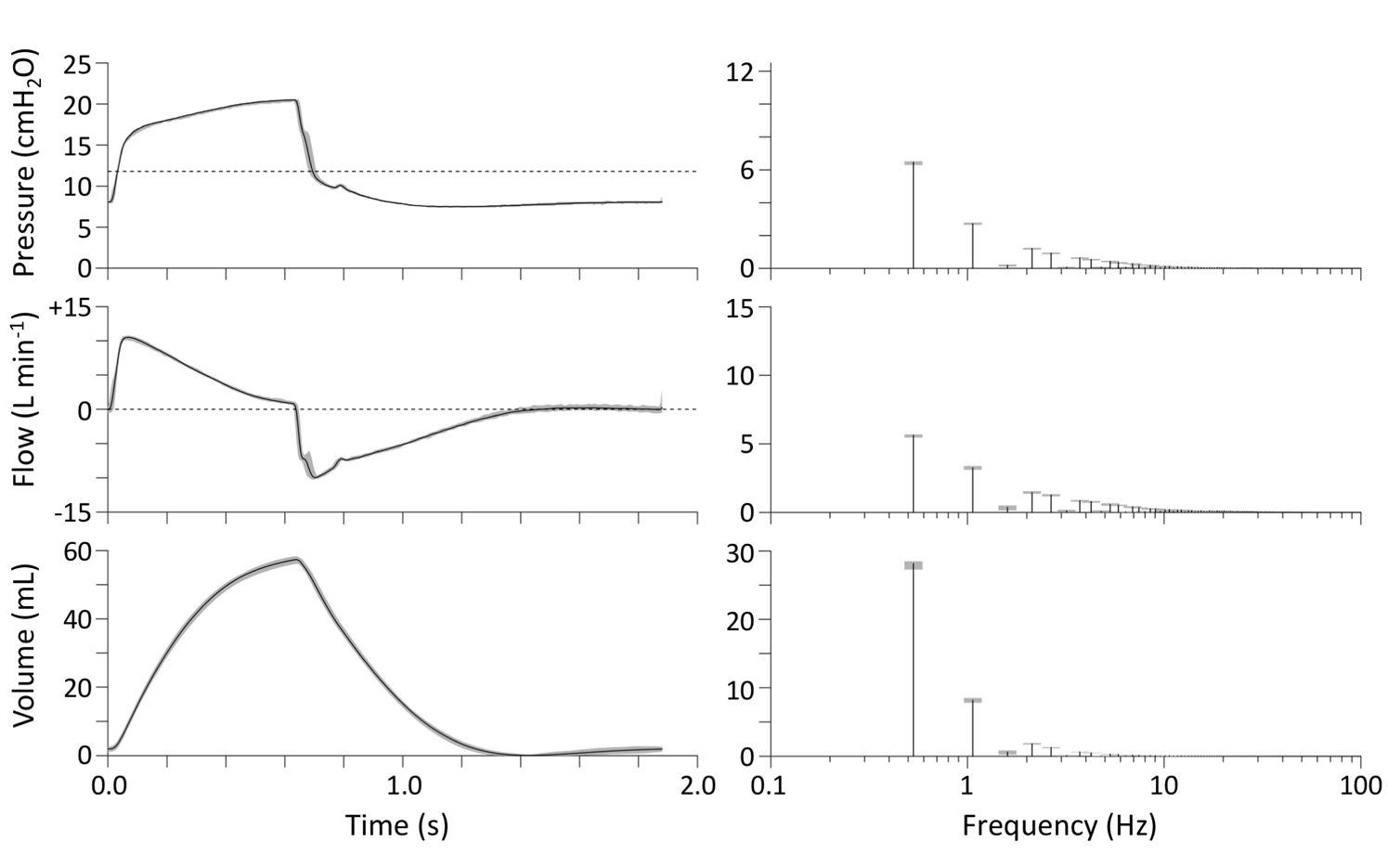
**

**Supplementary Figure S-1.** Ventilator waveforms measured at the airway opening during conventional mechanical ventilation (CMV). The discrete Fourier Transform was used to obtain the harmonic frequency content in each waveform over 1.875-second intervals. Each plot shows the average (solid black line) and range (gray shaded) over 60 breaths. Dotted lines indicate mean airway pressure and zero flow.


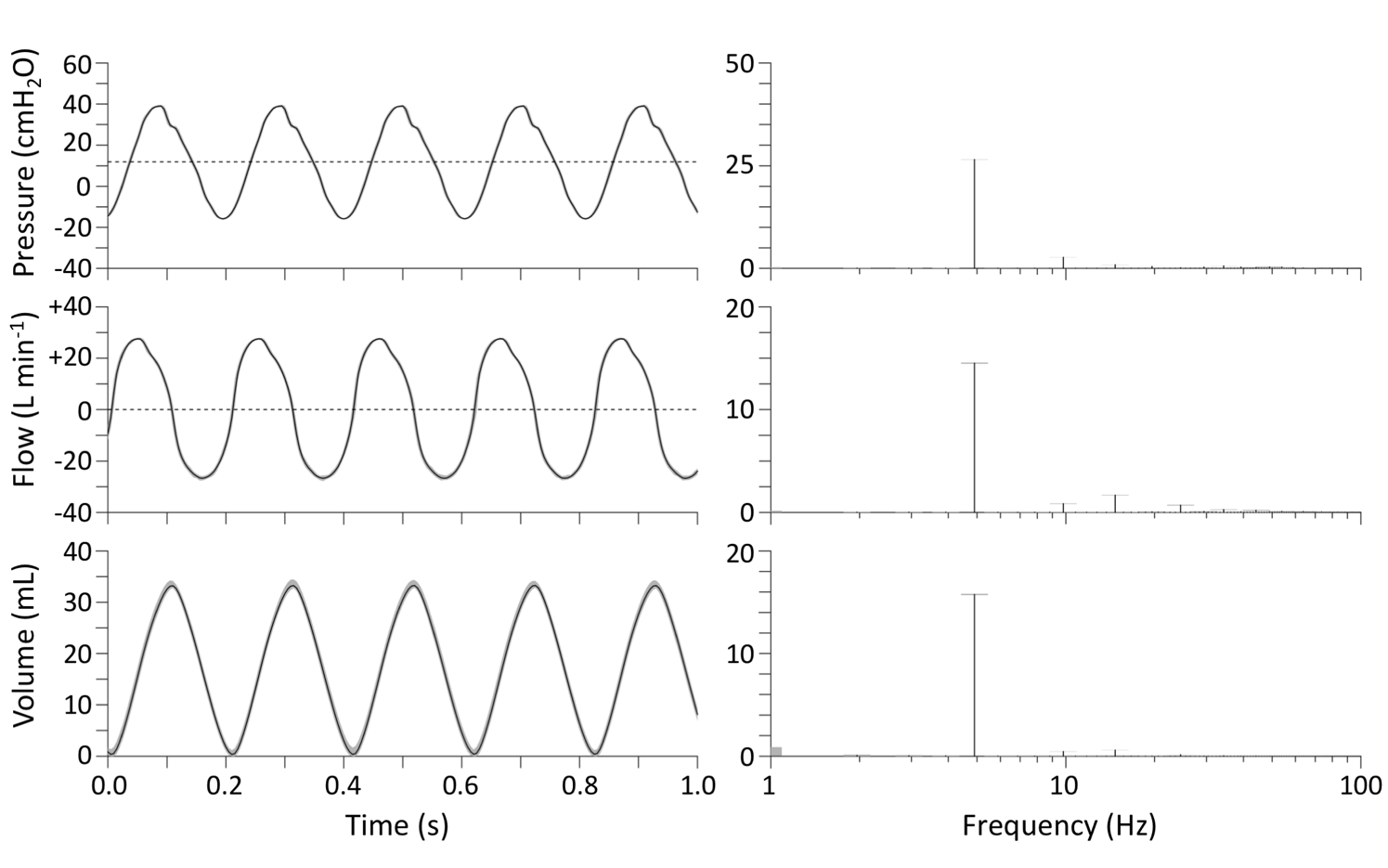


**Supplementary Figure S-2.** Ventilator waveforms measured at the airway opening during high-frequency oscillatory ventilation (HFOV). The discrete Fourier Transform was used to obtain the harmonic frequency content in each waveform over 1-second intervals. Each plot shows the average (solid black line) and range (gray shaded) over 74 seconds. Dotted lines indicate mean airway pressure and zero.

**
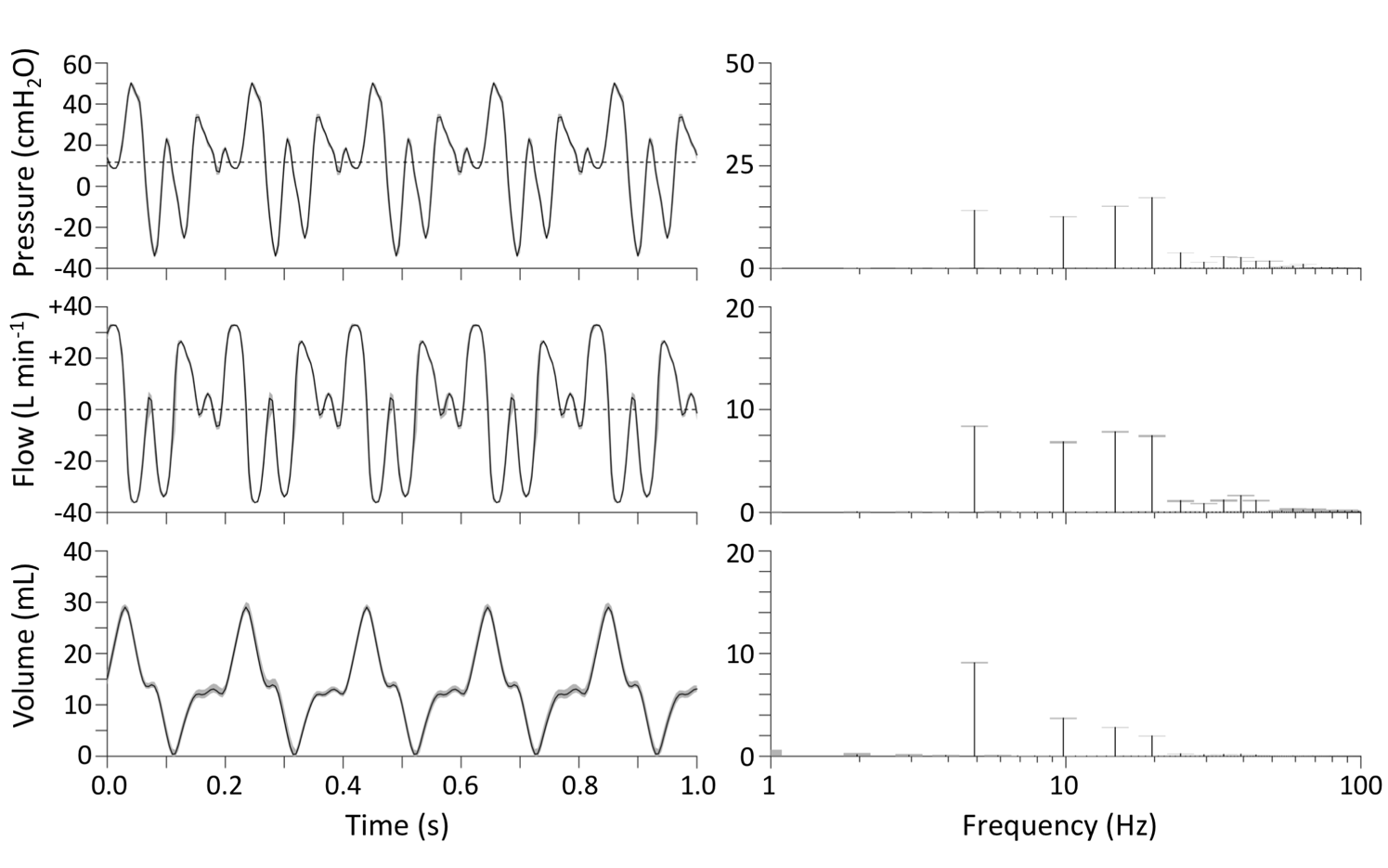
**

**Supplementary Figure S-3.** Ventilator waveforms measured at the airway opening during multi-frequency oscillatory ventilation (MFOV). The discrete Fourier Transform was used to obtain the harmonic frequency content in each waveform over 1-second intervals. Each plot shows the average (solid black line) and range (gray shaded) over 46 seconds. Dotted lines indicate mean airway pressure and zero flow.


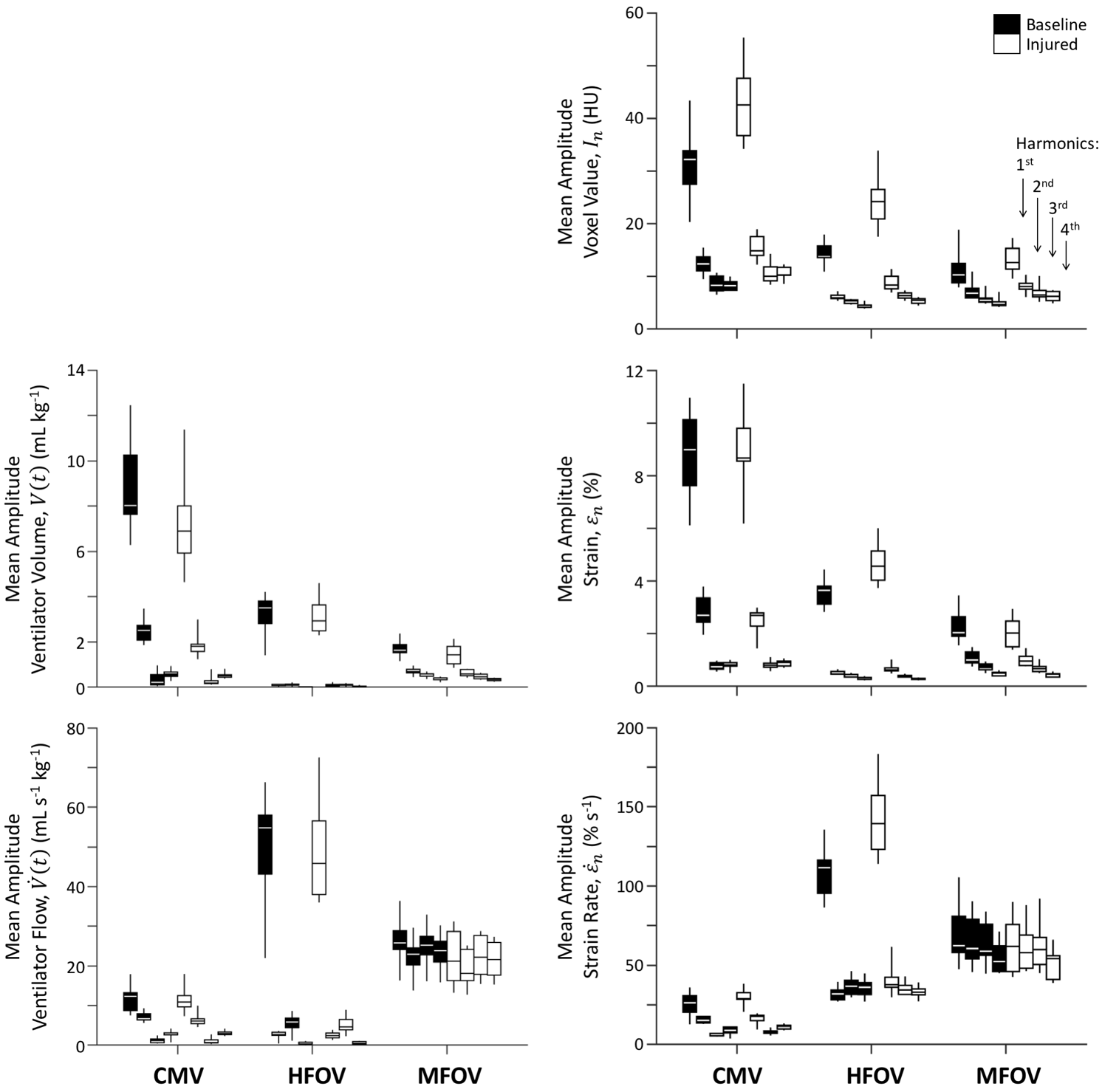


**Supplementary Figure S-4.** Frequency-domain representations of (left side) ventilator-based volume and flow at the airway opening, as well as (right side) image registration-based regional ventilation measures for intratidal changes in aeration (i.e., voxel value), volumetric strain, and volumetric strain rate. Amplitudes at the first four harmonics of the discrete Fourier transform are provided, grouped for each condition from left to right. Mean amplitude throughout the lung mask is shown for each variable, represented by box plots showing minimum, maximum, and quartiles across subjects under baseline (black) and injured (white) conditions, during conventional mechanical ventilation (CMV), high-frequency oscillatory ventilation (HFOV) and multi-frequency oscillatory ventilation (MFOV).
